# 4,4,6a,6b,11,12,14b-Heptamethyl-16-oxo-1,2,3,4,4a,5,6,6a,6b,7,8,9,10,11,12,12a,14a,14b-octa­deca­hydro-12b,8a-(epoxy­methano)­picen-3-yl acetate

**DOI:** 10.1107/S1600536813007253

**Published:** 2013-03-23

**Authors:** Mohammad Nisar, Sajid Ali, M. Nawaz Tahir, Bashir Ahmad, Shahid Hameed

**Affiliations:** a University of Peshawar, Institute of Chemical Sciences, Peshawar 25120, Pakistan; b University of Sargodha, Department of Physics, Sargodha, Pakistan; c University of Peshawar, Centre of Biotechnology and Microbiology, Peshawar 25120, Pakistan; d Quaid-i-Azam University, Department of Chemistry, Islamabad, Pakistan

## Abstract

The title compound, C_32_H_48_O_4_, which was extracted from the bark of *Rhododendron arboreum*, consists of five fused rings to which an acetate and seven methyl groups are attached. The *A*, *D* and *E* rings adopt chair conformations, the *B* ring is in a distorted chair and the C ring is in a half-chair conformation. The five-membered ring formed by the lactone group, which bridges from the *A*/*B* to the *B*/*C* ring junctions, is an approximate envelope with the C atom of the methyne group as the flap [displacement from the other four atoms = 0.753 (2) Å]. There are no identified directional inter­actions in the crystal structure.

## Related literature

For a related crystal structure, see: El-Seedi *et al.* (1994 ▶). For puckering parameters, see: Cremer & Pople (1975) ▶.
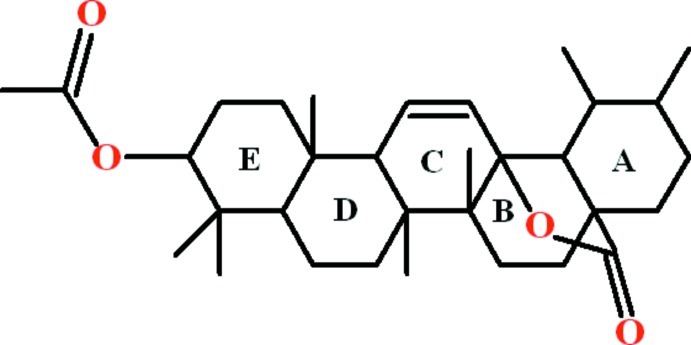


## Experimental

### 

#### Crystal data

C_32_H_48_O_4_
*M* *_r_* = 496.70
Monoclinic, 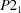

*a* = 13.7309 (8) Å
*b* = 6.9177 (4) Å
*c* = 14.8539 (9) Å
β = 90.943 (2)°
*V* = 1410.73 (14) Å^3^
*Z* = 2
Mo *K*α radiation
μ = 0.08 mm^−1^
*T* = 296 K
0.35 × 0.20 × 0.18 mm

#### Data collection

Bruker Kappa APEXII CCD diffractometer
Absorption correction: multi-scan (*SADABS*; Bruker, 2005 ▶) *T*_min_ = 0.975, *T*_max_ = 0.987
11401 measured reflections
2856 independent reflections
2261 reflections with *I* > 2σ(*I*)
*R*_int_ = 0.029

#### Refinement

*R*[*F* ^2^ > 2σ(*F* ^2^)] = 0.041
*wR*(*F* ^2^) = 0.101
*S* = 1.03
2856 reflections
333 parameters
1 restraint
H-atom parameters constrained
Δρ_max_ = 0.15 e Å^−3^
Δρ_min_ = −0.15 e Å^−3^

### 

Data collection: *APEX2* (Bruker, 2009 ▶); cell refinement: *SAINT* (Bruker, 2009 ▶); data reduction: *SAINT*; program(s) used to solve structure: *SHELXS97* (Sheldrick, 2008 ▶); program(s) used to refine structure: *SHELXL97* (Sheldrick, 2008 ▶); molecular graphics: *ORTEP-3 for Windows* (Farrugia, 2012 ▶) and *PLATON* (Spek, 2009 ▶); software used to prepare material for publication: *WinGX* (Farrugia, 2012 ▶) and *PLATON*.

## Supplementary Material

Crystal structure: contains datablock(s) global, I. DOI: 10.1107/S1600536813007253/hb7057sup1.cif

Structure factors: contains datablock(s) I. DOI: 10.1107/S1600536813007253/hb7057Isup2.hkl

Supplementary material file. DOI: 10.1107/S1600536813007253/hb7057Isup3.cml

Additional supplementary materials:  crystallographic information; 3D view; checkCIF report

## Figures and Tables

**Fig. 1.**
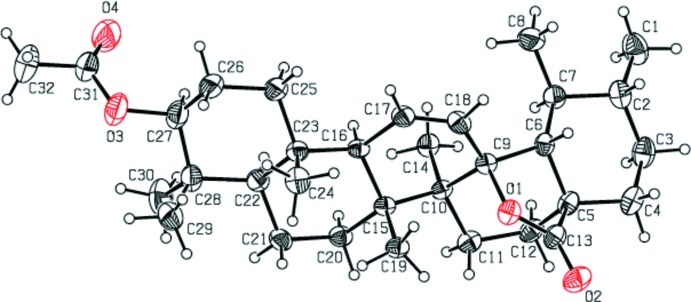
View of the title compound with displacement ellipsoids drawn at the 50% probability level.

## supplementary crystallographic information

### Comment

The title compound (I), (Fig. 1) has been extracted from the bark of *Rhododendron arboreum* collected in February 2011 from Butal, Hazar division, Pakistan.

The crystal structures of 3β,13β-13,28-Epoxy-3-acetoxy-11oleanene (El-Seedi *et al.*, 1994) extracted from the bark of *Minquartia guianensis* has been published which is related to (I).

In (I), five six-membered rings A (C2–C7), B (C5/C6/C9—C12), C (C9/C10/C15—C18), D (C15/C16/C20—C23) and E (C22/C23/C25—C28) are fused to each other. Seven methyl groups are attached at different positions. A carboxylate group is fused over ring A & B. One acetate group is also attached at the terminal ring E. The rings A, B, C, D and E are confirmed by different puckering parameters (Cremer & Pople, 1975). The puckering amplitude Q for rings A, B, C, D and E have values of 0.558 (3), 0.623 (3), 0.557 (3), 0.574 (3) & 0.561 (3) Å, θ for rings A, B, C, D and E have values of 3.6 (3), 164.3 (3), 49.3 (3), 172.3 (3) & 2.7 (3)°, φ for rings A, B, C, D and E have values of 323 (5), 230.3 (9), 102.6 (4), 359 (2) & 184 (7)°, respectively. The acetate group F (O3/O4/C31/C32) is planar with r. m. s. deviation of 0.0021 Å. It is oriented at a dihedral angle of 72.36 (0.15) ° with the plane of (C22/C25/C26/C28).

### Experimental

The dried and crushed barks of *Rhododendron arboreum* (5 kg) were subjected to cold extraction with methanol (MeOH). The MeOH extract (0.3 kg) was suspended in water and successively partitioned with n-hexane, CHCl_3_, EtOAc and butanol (BuOH). The CHCl_3_ fraction (15 g) was subjected to column chromatography on silica gel. The column was first eluted with n-hexane: CHC*L*_3_ (100:0 → 0:100) as solvent system. A total of 23 fractions, SF-1 to SF-23 were obtained based on TLC profiles. On further purification of fraction SF18 through pencil column colourless needles of (I) were obtained. Yield: 10 mg.

### Refinement

Anomanous dispersion was negligible and the absolute structure of (I) is indeterminate based on the present refinement. The H-atoms were positioned geometrically at C—H = 0.96—0.98 Å and included in the refinement as riding with *U*_iso_(H) = *xU*_eq_(C), where *x* = 1.5 for metyl H-atoms and *x* = 1.2 for all other H-atoms.

### Crystal data

**Table:**

C_32_H_48_O_4_	*F*(000) = 544
*M**_r_* = 496.70	*D*_x_ = 1.169 Mg m^−^^3^
Monoclinic, *P*2_1_	Mo *K*α radiation, λ = 0.71073 Å
Hall symbol: P 2yb	Cell parameters from 2261 reflections
*a* = 13.7309 (8) Å	θ = 2.7–25.3°
*b* = 6.9177 (4) Å	µ = 0.08 mm^−^^1^
*c* = 14.8539 (9) Å	*T* = 296 K
β = 90.943 (2)°	Needle, colorless
*V* = 1410.73 (14) Å^3^	0.35 × 0.20 × 0.18 mm
*Z* = 2	

### Data collection

**Table:**

Bruker Kappa APEXII CCD diffractometer	2856 independent reflections
Radiation source: fine-focus sealed tube	2261 reflections with *I* > 2σ(*I*)
Graphite monochromator	*R*_int_ = 0.029
Detector resolution: 8.10 pixels mm^-1^	θ_max_ = 25.5°, θ_min_ = 2.7°
ω scans	*h* = −15→16
Absorption correction: multi-scan (*SADABS*; Bruker, 2005)	*k* = −8→5
*T*_min_ = 0.975, *T*_max_ = 0.987	*l* = −17→17
11401 measured reflections	

### Refinement

**Table:**

Refinement on *F*^2^	Primary atom site location: structure-invariant direct methods
Least-squares matrix: full	Secondary atom site location: difference Fourier map
*R*[*F*^2^ > 2σ(*F*^2^)] = 0.041	Hydrogen site location: inferred from neighbouring sites
*wR*(*F*^2^) = 0.101	H-atom parameters constrained
*S* = 1.03	*w* = 1/[σ^2^(*F*_o_^2^) + (0.0488*P*)^2^ + 0.1608*P*] where *P* = (*F*_o_^2^ + 2*F*_c_^2^)/3
2856 reflections	(Δ/σ)_max_ < 0.001
333 parameters	Δρ_max_ = 0.15 e Å^−^^3^
1 restraint	Δρ_min_ = −0.15 e Å^−^^3^

### Special details

**Table:**

Geometry. Bond distances, angles *etc*. have been calculated using the rounded fractional coordinates. All su's are estimated from the variances of the (full) variance-covariance matrix. The cell e.s.d.'s are taken into account in the estimation of distances, angles and torsion angles
Refinement. Refinement of *F*^2^ against ALL reflections. The weighted *R*-factor *wR* and goodness of fit *S* are based on *F*^2^, conventional *R*-factors *R* are based on *F*, with *F* set to zero for negative *F*^2^. The threshold expression of *F*^2^ > σ(*F*^2^) is used only for calculating *R*-factors(gt) *etc*. and is not relevant to the choice of reflections for refinement. *R*-factors based on *F*^2^ are statistically about twice as large as those based on *F*, and *R*- factors based on ALL data will be even larger.

### Fractional atomic coordinates and isotropic or equivalent isotropic displacement parameters (Å^2^)

**Table:**

	*x*	*y*	*z*	*U*_iso_*/*U*_eq_	
O1	0.32780 (13)	0.9669 (3)	0.29373 (12)	0.0464 (6)	
O2	0.45050 (16)	1.0954 (3)	0.21754 (15)	0.0664 (8)	
O3	0.05842 (19)	0.5882 (4)	0.84926 (15)	0.0817 (10)	
O4	0.0088 (2)	0.2830 (5)	0.83569 (19)	0.1098 (14)	
C1	0.2961 (3)	0.2682 (6)	0.0396 (2)	0.0783 (14)	
C2	0.3127 (2)	0.4699 (5)	0.08018 (18)	0.0571 (10)	
C3	0.4184 (2)	0.5303 (5)	0.0680 (2)	0.0641 (11)	
C4	0.4413 (2)	0.7319 (5)	0.1039 (2)	0.0610 (11)	
C5	0.40994 (19)	0.7503 (4)	0.20146 (18)	0.0450 (9)	
C6	0.30424 (18)	0.6838 (4)	0.21412 (16)	0.0407 (8)	
C7	0.2829 (2)	0.4786 (4)	0.18059 (18)	0.0478 (9)	
C8	0.1764 (2)	0.4236 (6)	0.1928 (2)	0.0706 (12)	
C9	0.28636 (17)	0.7675 (4)	0.30996 (16)	0.0374 (8)	
C10	0.34669 (17)	0.6733 (4)	0.38810 (16)	0.0349 (8)	
C11	0.45584 (18)	0.6933 (4)	0.36732 (17)	0.0457 (9)	
C12	0.47923 (19)	0.6465 (4)	0.26904 (18)	0.0495 (10)	
C13	0.4016 (2)	0.9561 (4)	0.23483 (18)	0.0488 (9)	
C14	0.3239 (2)	0.4550 (4)	0.39160 (17)	0.0442 (8)	
C15	0.31827 (17)	0.7735 (4)	0.48074 (16)	0.0357 (8)	
C16	0.20947 (17)	0.7223 (3)	0.49750 (16)	0.0383 (8)	
C17	0.14878 (18)	0.7802 (5)	0.41643 (18)	0.0462 (9)	
C18	0.18247 (18)	0.7978 (4)	0.33399 (18)	0.0463 (9)	
C19	0.3337 (2)	0.9951 (3)	0.47758 (18)	0.0441 (9)	
C20	0.38119 (18)	0.6971 (4)	0.56019 (17)	0.0454 (9)	
C21	0.34095 (18)	0.7519 (5)	0.65228 (17)	0.0495 (10)	
C22	0.23627 (19)	0.6818 (4)	0.66293 (18)	0.0456 (9)	
C23	0.16692 (18)	0.7774 (4)	0.59105 (18)	0.0423 (8)	
C24	0.1520 (2)	0.9982 (4)	0.6012 (2)	0.0558 (11)	
C25	0.0663 (2)	0.6807 (5)	0.59872 (19)	0.0583 (10)	
C26	0.0281 (2)	0.6833 (6)	0.6953 (2)	0.0675 (11)	
C27	0.0994 (3)	0.5862 (5)	0.7590 (2)	0.0655 (11)	
C28	0.2011 (2)	0.6766 (5)	0.76271 (19)	0.0574 (11)	
C29	0.2007 (3)	0.8748 (6)	0.8091 (2)	0.0770 (16)	
C30	0.2685 (3)	0.5399 (7)	0.8174 (2)	0.0900 (16)	
C31	0.0121 (3)	0.4303 (7)	0.8771 (2)	0.0742 (14)	
C32	−0.0336 (3)	0.4631 (8)	0.9671 (2)	0.1036 (19)	
H1A	0.31578	0.26819	−0.02216	0.1174*	
H1B	0.22830	0.23553	0.04261	0.1174*	
H1C	0.33388	0.17490	0.07283	0.1174*	
H2	0.27151	0.56130	0.04654	0.0684*	
H3A	0.43318	0.52603	0.00442	0.0769*	
H3B	0.46032	0.43764	0.09863	0.0769*	
H4A	0.51065	0.75615	0.10003	0.0733*	
H4B	0.40742	0.82764	0.06737	0.0733*	
H6	0.26583	0.76721	0.17375	0.0488*	
H7	0.32370	0.38788	0.21507	0.0573*	
H8A	0.13590	0.50437	0.15519	0.1061*	
H8B	0.15916	0.44118	0.25459	0.1061*	
H8C	0.16697	0.29077	0.17610	0.1061*	
H11A	0.47650	0.82443	0.38058	0.0548*	
H11B	0.49275	0.60705	0.40647	0.0548*	
H12A	0.47438	0.50797	0.25986	0.0595*	
H12B	0.54579	0.68467	0.25724	0.0595*	
H14A	0.36235	0.38834	0.34798	0.0662*	
H14B	0.25605	0.43455	0.37834	0.0662*	
H14C	0.33927	0.40616	0.45065	0.0662*	
H16	0.20795	0.58066	0.49676	0.0460*	
H17	0.08305	0.80536	0.42502	0.0554*	
H18	0.13867	0.83109	0.28815	0.0555*	
H19A	0.33521	1.04567	0.53778	0.0661*	
H19B	0.28112	1.05391	0.44409	0.0661*	
H19C	0.39420	1.02319	0.44892	0.0661*	
H20A	0.38526	0.55736	0.55608	0.0545*	
H20B	0.44664	0.74832	0.55529	0.0545*	
H21A	0.38173	0.69537	0.69928	0.0594*	
H21B	0.34304	0.89125	0.65922	0.0594*	
H22	0.23849	0.54547	0.64517	0.0547*	
H24A	0.13717	1.05359	0.54333	0.0837*	
H24B	0.21051	1.05555	0.62533	0.0837*	
H24C	0.09918	1.02218	0.64120	0.0837*	
H25A	0.02015	0.74730	0.55946	0.0699*	
H25B	0.07070	0.54785	0.57835	0.0699*	
H26A	0.01795	0.81592	0.71418	0.0809*	
H26B	−0.03406	0.61680	0.69708	0.0809*	
H27	0.10620	0.45116	0.74024	0.0784*	
H29A	0.14881	0.95220	0.78418	0.1155*	
H29B	0.26180	0.93833	0.79966	0.1155*	
H29C	0.19106	0.85794	0.87249	0.1155*	
H30A	0.24031	0.51464	0.87495	0.1345*	
H30B	0.33106	0.59959	0.82595	0.1345*	
H30C	0.27590	0.42051	0.78530	0.1345*	
H32A	−0.06580	0.34703	0.98592	0.1552*	
H32B	−0.08018	0.56618	0.96236	0.1552*	
H32C	0.01602	0.49687	1.01063	0.1552*	

### Atomic displacement parameters (Å^2^)

**Table:**

	*U*^11^	*U*^22^	*U*^33^	*U*^12^	*U*^13^	*U*^23^
O1	0.0564 (11)	0.0303 (9)	0.0526 (11)	0.0062 (9)	0.0044 (9)	0.0073 (8)
O2	0.0744 (15)	0.0429 (12)	0.0820 (16)	−0.0120 (11)	0.0050 (12)	0.0171 (11)
O3	0.112 (2)	0.0720 (17)	0.0624 (14)	−0.0162 (15)	0.0373 (13)	−0.0018 (12)
O4	0.150 (3)	0.095 (2)	0.085 (2)	−0.049 (2)	0.0222 (18)	0.0005 (19)
C1	0.109 (3)	0.069 (2)	0.057 (2)	0.005 (2)	0.0036 (19)	−0.0152 (18)
C2	0.074 (2)	0.0529 (17)	0.0445 (16)	0.0090 (17)	0.0024 (14)	0.0008 (14)
C3	0.078 (2)	0.061 (2)	0.0540 (19)	0.0142 (18)	0.0183 (16)	0.0011 (15)
C4	0.063 (2)	0.062 (2)	0.0585 (19)	0.0050 (16)	0.0183 (15)	0.0111 (15)
C5	0.0459 (16)	0.0402 (15)	0.0493 (16)	0.0042 (13)	0.0102 (12)	0.0075 (12)
C6	0.0441 (15)	0.0396 (14)	0.0383 (14)	0.0060 (12)	0.0017 (11)	0.0068 (12)
C7	0.0563 (17)	0.0407 (15)	0.0463 (15)	−0.0001 (14)	0.0018 (12)	−0.0007 (12)
C8	0.066 (2)	0.078 (2)	0.068 (2)	−0.0183 (18)	0.0033 (16)	−0.0197 (18)
C9	0.0390 (14)	0.0291 (12)	0.0440 (15)	0.0047 (11)	−0.0012 (11)	0.0036 (11)
C10	0.0314 (13)	0.0286 (12)	0.0445 (14)	0.0015 (11)	−0.0006 (11)	0.0063 (11)
C11	0.0377 (15)	0.0431 (15)	0.0562 (17)	0.0047 (12)	−0.0002 (12)	0.0073 (13)
C12	0.0388 (15)	0.0467 (17)	0.0634 (18)	0.0045 (13)	0.0098 (13)	0.0090 (14)
C13	0.0556 (18)	0.0392 (15)	0.0516 (16)	0.0006 (14)	0.0035 (14)	0.0118 (13)
C14	0.0547 (16)	0.0307 (13)	0.0472 (15)	0.0032 (13)	0.0022 (12)	0.0043 (12)
C15	0.0351 (13)	0.0267 (12)	0.0451 (14)	0.0007 (11)	−0.0040 (11)	0.0042 (11)
C16	0.0363 (14)	0.0322 (14)	0.0463 (15)	−0.0022 (10)	−0.0036 (11)	0.0001 (11)
C17	0.0311 (13)	0.0579 (17)	0.0494 (17)	0.0049 (14)	−0.0014 (12)	−0.0036 (14)
C18	0.0381 (14)	0.0522 (17)	0.0482 (17)	0.0115 (13)	−0.0092 (12)	−0.0003 (13)
C19	0.0490 (16)	0.0301 (14)	0.0532 (16)	−0.0053 (12)	−0.0006 (12)	0.0002 (12)
C20	0.0398 (15)	0.0444 (15)	0.0518 (16)	−0.0001 (12)	−0.0077 (12)	0.0063 (13)
C21	0.0487 (16)	0.0545 (18)	0.0449 (16)	−0.0017 (14)	−0.0116 (12)	0.0038 (13)
C22	0.0516 (16)	0.0370 (14)	0.0482 (15)	−0.0017 (13)	−0.0006 (12)	0.0019 (12)
C23	0.0407 (15)	0.0401 (14)	0.0460 (15)	−0.0014 (13)	0.0016 (12)	−0.0018 (12)
C24	0.0584 (18)	0.0503 (19)	0.0589 (18)	0.0133 (15)	0.0042 (14)	−0.0026 (14)
C25	0.0456 (16)	0.075 (2)	0.0546 (18)	−0.0080 (16)	0.0097 (13)	−0.0088 (16)
C26	0.0585 (19)	0.083 (2)	0.0615 (19)	−0.0168 (19)	0.0159 (16)	−0.0089 (18)
C27	0.086 (2)	0.058 (2)	0.0533 (19)	−0.0095 (19)	0.0227 (17)	−0.0043 (16)
C28	0.070 (2)	0.0596 (19)	0.0428 (16)	−0.0005 (17)	0.0050 (14)	0.0030 (15)
C29	0.095 (3)	0.080 (3)	0.056 (2)	−0.015 (2)	0.0053 (18)	−0.0143 (19)
C30	0.105 (3)	0.108 (3)	0.057 (2)	0.019 (3)	0.0047 (19)	0.027 (2)
C31	0.080 (2)	0.085 (3)	0.058 (2)	−0.010 (2)	0.0121 (18)	0.015 (2)
C32	0.119 (3)	0.118 (4)	0.075 (3)	−0.002 (3)	0.038 (2)	0.023 (3)

### Geometric parameters (Å, º)

**Table:**

O1—C9	1.513 (3)	C3—H3A	0.9700
O1—C13	1.352 (3)	C3—H3B	0.9700
O2—C13	1.205 (3)	C4—H4A	0.9700
O3—C27	1.463 (4)	C4—H4B	0.9700
O3—C31	1.333 (5)	C6—H6	0.9800
O4—C31	1.191 (6)	C7—H7	0.9800
C1—C2	1.536 (5)	C8—H8A	0.9600
C2—C3	1.524 (4)	C8—H8B	0.9600
C2—C7	1.554 (4)	C8—H8C	0.9600
C3—C4	1.524 (5)	C11—H11A	0.9700
C4—C5	1.524 (4)	C11—H11B	0.9700
C5—C6	1.537 (4)	C12—H12A	0.9700
C5—C12	1.548 (4)	C12—H12B	0.9700
C5—C13	1.512 (4)	C14—H14A	0.9600
C6—C7	1.531 (4)	C14—H14B	0.9600
C6—C9	1.560 (3)	C14—H14C	0.9600
C7—C8	1.525 (4)	C16—H16	0.9800
C9—C10	1.558 (3)	C17—H17	0.9300
C9—C18	1.491 (3)	C18—H18	0.9300
C10—C11	1.541 (3)	C19—H19A	0.9600
C10—C14	1.543 (4)	C19—H19B	0.9600
C10—C15	1.595 (3)	C19—H19C	0.9600
C11—C12	1.534 (4)	C20—H20A	0.9700
C15—C16	1.559 (3)	C20—H20B	0.9700
C15—C19	1.548 (3)	C21—H21A	0.9700
C15—C20	1.544 (4)	C21—H21B	0.9700
C16—C17	1.507 (4)	C22—H22	0.9800
C16—C23	1.563 (4)	C24—H24A	0.9600
C17—C18	1.322 (4)	C24—H24B	0.9600
C20—C21	1.531 (4)	C24—H24C	0.9600
C21—C22	1.528 (4)	C25—H25A	0.9700
C22—C23	1.565 (4)	C25—H25B	0.9700
C22—C28	1.567 (4)	C26—H26A	0.9700
C23—C24	1.549 (4)	C26—H26B	0.9700
C23—C25	1.541 (4)	C27—H27	0.9800
C25—C26	1.536 (4)	C29—H29A	0.9600
C26—C27	1.508 (5)	C29—H29B	0.9600
C27—C28	1.530 (5)	C29—H29C	0.9600
C28—C29	1.535 (5)	C30—H30A	0.9600
C28—C30	1.545 (5)	C30—H30B	0.9600
C31—C32	1.503 (5)	C30—H30C	0.9600
C1—H1A	0.9600	C32—H32A	0.9600
C1—H1B	0.9600	C32—H32B	0.9600
C1—H1C	0.9600	C32—H32C	0.9600
C2—H2	0.9800		
			
C9—O1—C13	109.9 (2)	C5—C6—H6	104.00
C27—O3—C31	118.0 (3)	C7—C6—H6	104.00
C1—C2—C3	109.8 (3)	C9—C6—H6	104.00
C1—C2—C7	111.9 (3)	C2—C7—H7	109.00
C3—C2—C7	111.7 (2)	C6—C7—H7	109.00
C2—C3—C4	113.6 (3)	C8—C7—H7	109.00
C3—C4—C5	110.4 (2)	C7—C8—H8A	109.00
C4—C5—C6	111.9 (2)	C7—C8—H8B	110.00
C4—C5—C12	113.4 (2)	C7—C8—H8C	109.00
C4—C5—C13	114.5 (2)	H8A—C8—H8B	109.00
C6—C5—C12	110.7 (2)	H8A—C8—H8C	109.00
C6—C5—C13	99.5 (2)	H8B—C8—H8C	110.00
C12—C5—C13	105.9 (2)	C10—C11—H11A	109.00
C5—C6—C7	114.4 (2)	C10—C11—H11B	109.00
C5—C6—C9	99.4 (2)	C12—C11—H11A	109.00
C7—C6—C9	127.5 (2)	C12—C11—H11B	109.00
C2—C7—C6	107.2 (2)	H11A—C11—H11B	108.00
C2—C7—C8	111.9 (2)	C5—C12—H12A	109.00
C6—C7—C8	111.8 (2)	C5—C12—H12B	109.00
O1—C9—C6	97.34 (18)	C11—C12—H12A	109.00
O1—C9—C10	107.66 (19)	C11—C12—H12B	109.00
O1—C9—C18	105.9 (2)	H12A—C12—H12B	108.00
C6—C9—C10	115.8 (2)	C10—C14—H14A	109.00
C6—C9—C18	115.9 (2)	C10—C14—H14B	110.00
C10—C9—C18	112.3 (2)	C10—C14—H14C	109.00
C9—C10—C11	108.7 (2)	H14A—C14—H14B	109.00
C9—C10—C14	109.2 (2)	H14A—C14—H14C	109.00
C9—C10—C15	109.1 (2)	H14B—C14—H14C	109.00
C11—C10—C14	107.0 (2)	C15—C16—H16	104.00
C11—C10—C15	112.7 (2)	C17—C16—H16	104.00
C14—C10—C15	110.1 (2)	C23—C16—H16	104.00
C10—C11—C12	113.0 (2)	C16—C17—H17	118.00
C5—C12—C11	112.5 (2)	C18—C17—H17	118.00
O1—C13—O2	121.4 (3)	C9—C18—H18	118.00
O1—C13—C5	109.0 (2)	C17—C18—H18	118.00
O2—C13—C5	129.6 (3)	C15—C19—H19A	109.00
C10—C15—C16	106.72 (19)	C15—C19—H19B	109.00
C10—C15—C19	111.6 (2)	C15—C19—H19C	110.00
C10—C15—C20	111.6 (2)	H19A—C19—H19B	109.00
C16—C15—C19	111.2 (2)	H19A—C19—H19C	110.00
C16—C15—C20	109.0 (2)	H19B—C19—H19C	109.00
C19—C15—C20	106.7 (2)	C15—C20—H20A	109.00
C15—C16—C17	109.3 (2)	C15—C20—H20B	109.00
C15—C16—C23	117.36 (19)	C21—C20—H20A	109.00
C17—C16—C23	115.8 (2)	C21—C20—H20B	109.00
C16—C17—C18	124.6 (2)	H20A—C20—H20B	108.00
C9—C18—C17	124.0 (2)	C20—C21—H21A	109.00
C15—C20—C21	113.1 (2)	C20—C21—H21B	109.00
C20—C21—C22	111.6 (2)	C22—C21—H21A	109.00
C21—C22—C23	111.0 (2)	C22—C21—H21B	109.00
C21—C22—C28	114.2 (2)	H21A—C21—H21B	108.00
C23—C22—C28	117.5 (2)	C21—C22—H22	104.00
C16—C23—C22	105.7 (2)	C23—C22—H22	104.00
C16—C23—C24	112.3 (2)	C28—C22—H22	104.00
C16—C23—C25	108.0 (2)	C23—C24—H24A	109.00
C22—C23—C24	115.5 (2)	C23—C24—H24B	109.00
C22—C23—C25	107.6 (2)	C23—C24—H24C	109.00
C24—C23—C25	107.5 (2)	H24A—C24—H24B	109.00
C23—C25—C26	112.6 (2)	H24A—C24—H24C	110.00
C25—C26—C27	110.7 (3)	H24B—C24—H24C	109.00
O3—C27—C26	108.4 (3)	C23—C25—H25A	109.00
O3—C27—C28	109.1 (3)	C23—C25—H25B	109.00
C26—C27—C28	115.0 (3)	C26—C25—H25A	109.00
C22—C28—C27	105.7 (2)	C26—C25—H25B	109.00
C22—C28—C29	114.1 (3)	H25A—C25—H25B	108.00
C22—C28—C30	108.7 (2)	C25—C26—H26A	110.00
C27—C28—C29	111.8 (3)	C25—C26—H26B	110.00
C27—C28—C30	107.9 (3)	C27—C26—H26A	110.00
C29—C28—C30	108.5 (3)	C27—C26—H26B	110.00
O3—C31—O4	123.7 (3)	H26A—C26—H26B	108.00
O3—C31—C32	111.1 (4)	O3—C27—H27	108.00
O4—C31—C32	125.1 (4)	C26—C27—H27	108.00
C2—C1—H1A	109.00	C28—C27—H27	108.00
C2—C1—H1B	109.00	C28—C29—H29A	109.00
C2—C1—H1C	109.00	C28—C29—H29B	110.00
H1A—C1—H1B	109.00	C28—C29—H29C	109.00
H1A—C1—H1C	109.00	H29A—C29—H29B	109.00
H1B—C1—H1C	110.00	H29A—C29—H29C	109.00
C1—C2—H2	108.00	H29B—C29—H29C	110.00
C3—C2—H2	108.00	C28—C30—H30A	109.00
C7—C2—H2	108.00	C28—C30—H30B	109.00
C2—C3—H3A	109.00	C28—C30—H30C	109.00
C2—C3—H3B	109.00	H30A—C30—H30B	109.00
C4—C3—H3A	109.00	H30A—C30—H30C	109.00
C4—C3—H3B	109.00	H30B—C30—H30C	110.00
H3A—C3—H3B	108.00	C31—C32—H32A	109.00
C3—C4—H4A	110.00	C31—C32—H32B	110.00
C3—C4—H4B	110.00	C31—C32—H32C	109.00
C5—C4—H4A	110.00	H32A—C32—H32B	109.00
C5—C4—H4B	110.00	H32A—C32—H32C	109.00
H4A—C4—H4B	108.00	H32B—C32—H32C	109.00
			
C13—O1—C9—C6	−32.6 (2)	C14—C10—C11—C12	74.0 (3)
C13—O1—C9—C10	87.5 (2)	C15—C10—C11—C12	−164.9 (2)
C13—O1—C9—C18	−152.2 (2)	C9—C10—C15—C16	65.3 (2)
C9—O1—C13—O2	−174.0 (2)	C9—C10—C15—C19	−56.4 (3)
C9—O1—C13—C5	4.4 (3)	C9—C10—C15—C20	−175.7 (2)
C31—O3—C27—C26	97.1 (4)	C11—C10—C15—C16	−174.0 (2)
C31—O3—C27—C28	−136.9 (3)	C11—C10—C15—C19	64.3 (3)
C27—O3—C31—O4	6.1 (6)	C11—C10—C15—C20	−55.0 (3)
C27—O3—C31—C32	−174.5 (3)	C14—C10—C15—C16	−54.5 (2)
C1—C2—C3—C4	178.3 (2)	C14—C10—C15—C19	−176.2 (2)
C7—C2—C3—C4	−57.0 (3)	C14—C10—C15—C20	64.5 (3)
C1—C2—C7—C6	179.0 (2)	C10—C11—C12—C5	48.1 (3)
C1—C2—C7—C8	−58.1 (3)	C10—C15—C16—C17	−54.2 (3)
C3—C2—C7—C6	55.5 (3)	C10—C15—C16—C23	171.3 (2)
C3—C2—C7—C8	178.4 (3)	C19—C15—C16—C17	67.8 (3)
C2—C3—C4—C5	53.2 (3)	C19—C15—C16—C23	−66.7 (3)
C3—C4—C5—C6	−50.6 (3)	C20—C15—C16—C17	−174.9 (2)
C3—C4—C5—C12	75.5 (3)	C20—C15—C16—C23	50.6 (3)
C3—C4—C5—C13	−162.9 (2)	C10—C15—C20—C21	−166.3 (2)
C4—C5—C6—C7	54.9 (3)	C16—C15—C20—C21	−48.6 (3)
C4—C5—C6—C9	−166.2 (2)	C19—C15—C20—C21	71.5 (3)
C12—C5—C6—C7	−72.7 (3)	C15—C16—C17—C18	24.5 (4)
C12—C5—C6—C9	66.2 (3)	C23—C16—C17—C18	159.8 (3)
C13—C5—C6—C7	176.2 (2)	C15—C16—C23—C22	−54.8 (3)
C13—C5—C6—C9	−44.9 (2)	C15—C16—C23—C24	72.0 (3)
C4—C5—C12—C11	170.9 (2)	C15—C16—C23—C25	−169.7 (2)
C6—C5—C12—C11	−62.3 (3)	C17—C16—C23—C22	173.7 (2)
C13—C5—C12—C11	44.6 (3)	C17—C16—C23—C24	−59.6 (3)
C4—C5—C13—O1	145.8 (2)	C17—C16—C23—C25	58.7 (3)
C4—C5—C13—O2	−36.0 (4)	C16—C17—C18—C9	−1.6 (5)
C6—C5—C13—O1	26.3 (3)	C15—C20—C21—C22	56.5 (3)
C6—C5—C13—O2	−155.5 (3)	C20—C21—C22—C23	−61.7 (3)
C12—C5—C13—O1	−88.5 (2)	C20—C21—C22—C28	162.7 (2)
C12—C5—C13—O2	89.7 (3)	C21—C22—C23—C16	57.9 (3)
C5—C6—C7—C2	−55.5 (3)	C21—C22—C23—C24	−66.9 (3)
C5—C6—C7—C8	−178.5 (2)	C21—C22—C23—C25	173.1 (2)
C9—C6—C7—C2	179.4 (2)	C28—C22—C23—C16	−168.1 (2)
C9—C6—C7—C8	56.4 (3)	C28—C22—C23—C24	67.1 (3)
C5—C6—C9—O1	46.7 (2)	C28—C22—C23—C25	−52.9 (3)
C5—C6—C9—C10	−67.0 (3)	C21—C22—C28—C27	−175.0 (3)
C5—C6—C9—C18	158.3 (2)	C21—C22—C28—C29	61.8 (3)
C7—C6—C9—O1	177.7 (2)	C21—C22—C28—C30	−59.4 (4)
C7—C6—C9—C10	64.0 (3)	C23—C22—C28—C27	52.4 (3)
C7—C6—C9—C18	−70.7 (3)	C23—C22—C28—C29	−70.8 (3)
O1—C9—C10—C11	−50.0 (3)	C23—C22—C28—C30	168.0 (3)
O1—C9—C10—C14	−166.42 (19)	C16—C23—C25—C26	166.3 (3)
O1—C9—C10—C15	73.2 (2)	C22—C23—C25—C26	52.6 (3)
C6—C9—C10—C11	57.6 (3)	C24—C23—C25—C26	−72.4 (3)
C6—C9—C10—C14	−58.8 (3)	C23—C25—C26—C27	−57.1 (4)
C6—C9—C10—C15	−179.2 (2)	C25—C26—C27—O3	−178.5 (3)
C18—C9—C10—C11	−166.2 (2)	C25—C26—C27—C28	59.0 (4)
C18—C9—C10—C14	77.4 (3)	O3—C27—C28—C22	−176.2 (2)
C18—C9—C10—C15	−42.9 (3)	O3—C27—C28—C29	−51.5 (3)
O1—C9—C18—C17	−105.8 (3)	O3—C27—C28—C30	67.7 (3)
C6—C9—C18—C17	147.6 (3)	C26—C27—C28—C22	−54.1 (3)
C10—C9—C18—C17	11.5 (4)	C26—C27—C28—C29	70.5 (3)
C9—C10—C11—C12	−43.9 (3)	C26—C27—C28—C30	−170.2 (3)

## References

[bb1] Bruker (2005). *SADABS* Bruker AXS Inc., Madison, Wisconsin, USA.

[bb2] Bruker (2009). *APEX2* and *SAINT* Bruker AXS Inc., Madison, Wisconsin, USA.

[bb7] Cremer, D. & Pople, J. A. (1975). *J. Am. Chem. Soc.* **97**, 1354–1358.

[bb3] El-Seedi, H. R., Hazell, A. C. & Torssell, K. B. G. (1994). *Phytochemistry*, **35**, 1297–1299.

[bb4] Farrugia, L. J. (2012). *J. Appl. Cryst.* **45**, 849–854.

[bb5] Sheldrick, G. M. (2008). *Acta Cryst.* A**64**, 112–122. 10.1107/S0108767307043930 18156677

[bb6] Spek, A. L. (2009). *Acta Cryst.* D**65**, 148–155. 10.1107/S090744490804362X PMC2631630 19171970

